# 35 Using a New Quality Registry System to Improve Data Completeness for a National Burn Registry

**DOI:** 10.1093/jbcr/irac012.038

**Published:** 2022-03-23

**Authors:** Bart D Phillips, Palmer Q Bessey, Samuel P Mandell, Callie M Thompson, Matthew H Phillips, Kimberly Hoarle, Sara Higginson, Naiwei Hsu, Darryl A Pardon, Joan M Weber

**Affiliations:** BData, Inc, Edina, Minnesota; New Year-Presbyterian Hospital / Weill Cornell Medicine, New York, New York; UT Southwestern, Parkland Regional Burn Center, Dallas, Texas; University of Utah Health, Salt Lake City, Utah; BData, Inc, Apex, North Carolina; American Burn Association, Chicago, Illinois; UC San Diego Health Regional Burn Center, San Diego, California; Torrance Memorial Medical Center, Torrance, California; BData, Inc, Louisville, Kentucky; Shriners Hospitals for Children Boston, Boston, Massachusetts

## Abstract

**Introduction:**

The burn care community has demonstrated a long-standing commitment to quality of care, improved outcomes, and research by collecting and sharing clinical data through burn registries. The key to optimizing the value creation from these registries is data quality. In 2019 a new registry platform incorporating the latest, standardized, data definitions and sophisticated audit controls was piloted with 12 burn centers. The following year it was introduced to the broader burn care community along with a robust registrar education and training program. We sought to evaluate the effect of this new system on quality of data collection.

**Methods:**

We compared data from 27 centers that collected data on the new system against their data collected on prior registry systems. We analyzed 26 data elements, across four different variable categories (Admissions (n=6), Demographics (n=5), Injury (n=10), and Outcomes(n=5)) that have been consistently collected over time, for data completeness. A two-proportion z test was used to assess statistical significance of differences in data completeness rates.

**Results:**

The 27-center cohort entered data on 4,524 inpatient burn admissions between January 1, 2020 and June 30^th^ 2021 and 7,259 admissions between July 1^st^, 2019 and June 30^th^, 2020 in their legacy system. The data elements were harmonized to maximize longitudinal comparability.

When comparing data from the same centers but using different registry software (graphic 1) 16 of 26 variables profiled had a higher percentage of completeness in the new registry system (all statistically significant), 5 variables were statistically significantly lower, and 5 variables were not significantly different. Overall, this subset of data elements showed a 14% improvement in data completeness between the new and legacy systems (90% vs 75%).

**Conclusions:**

The development of a clinical registry requires significant commitment and leadership from the healthcare association and broader clinical community. The resources and effort that the burn community has expended to improve data quality has produced important gains in data quality with the introduction of a new, high-quality registry. The burn care community should continue to emphasize education, innovation, and collaboration to collect the highest quality data at the lowest burden from all burn centers.

